# Editorial: Advances and Perspective in bio-implants for commercialization

**DOI:** 10.3389/fbioe.2023.1306077

**Published:** 2023-10-17

**Authors:** Sarabjeet Singh Sidhu, Arnab Chanda, Mohamed Abdel-Hady Gepreel

**Affiliations:** ^1^ Mechanical Engineering Department, Sardar Beant Singh State University (Formerly known as Beant College of Engineering and Technology), Gurdaspur, Punjab, India; ^2^ Centre for Biomedical Engineering, Indian Institute of Technology (IIT), New Delhi, India; ^3^ Egypt-Japan University of Science and Technology (E-JUST), Alexandria, Egypt

**Keywords:** bioimplants, coating, technology translation, 3D printing, editorial

## Introduction

Bio-implants have undergone a transformative journey within the domain of medical science, through the intersection of medical and engineering fields. These remarkable hybrid approaches have opened new frontiers in healthcare. Over time, these bio-implants have undergone a profound evolution, seamlessly integrating cutting-edge technologies and materials. In this Research Topic, we embark on a captivating exploration of the exhilarating advancements and potential trajectories in the realm of bio-implants, with a keen emphasis on their commercialization. The Research Topic of articles in this issue aims to inspire recent developments made in the design and development of sustainable implants, ultimately propelling them towards commercial viability. The journey from innovation to commercialization represents a monumental leap and the articles in this Research Topic shed light on the extremely significant advances in this field of biomedical research in recent years.

**FIGURE 1:**
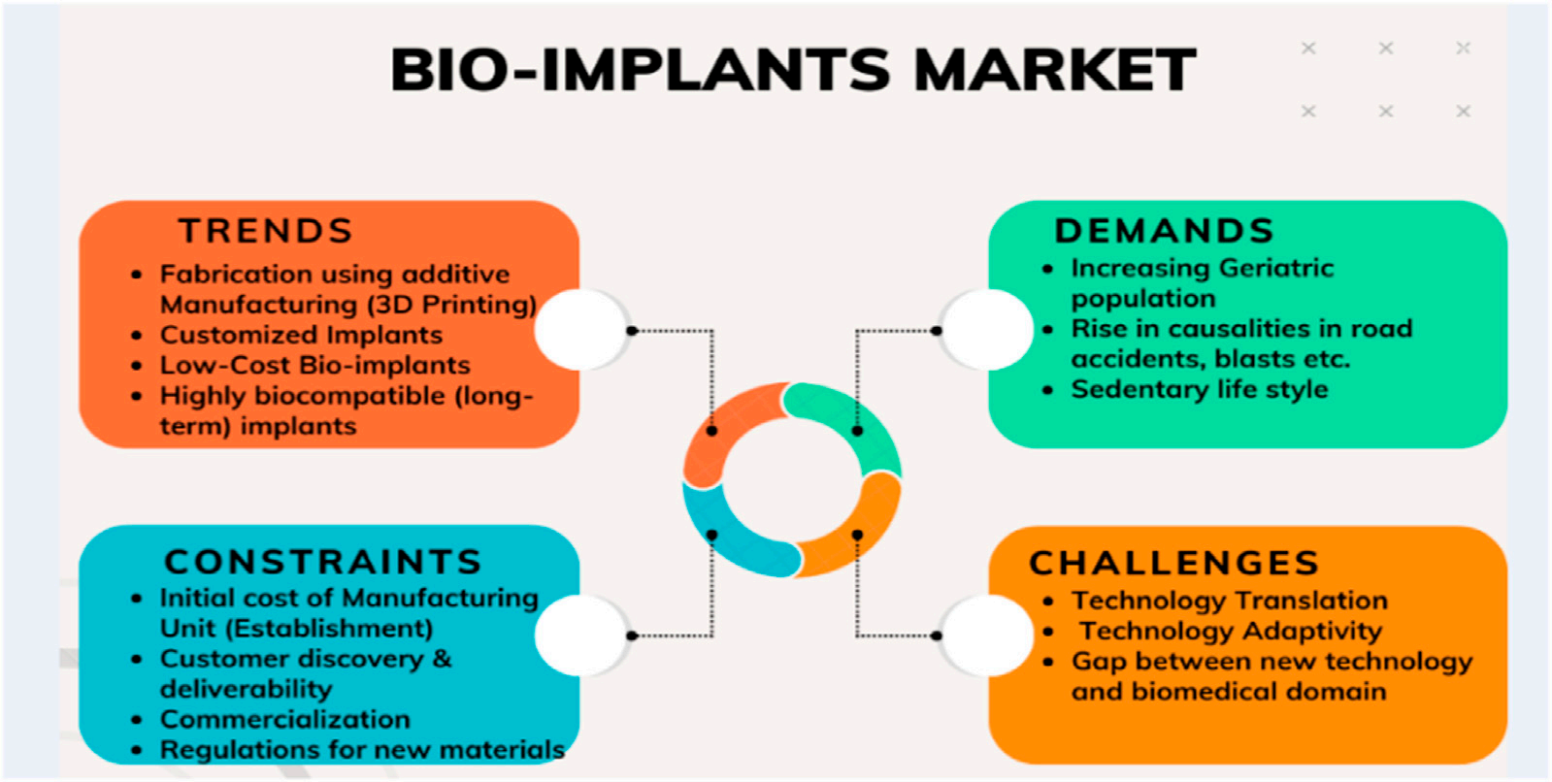
Global bio-implant market.

The landscape of emerging biomaterials has experienced a remarkable resurgence in recent decades, largely spurred by an unprecedented dedication to healthcare technologies and their journey toward commercialization. Metallic and ceramic biomaterials, owing to their exceptional mechanical robustness, have enjoyed widespread utilization as load-bearing implants and internal fixation devices. However, polymer-based and bio-composite materials are now emerging as strong contenders in the realm of soft tissue engineering. Despite the established trust in many alloys and soft materials for biomedical applications, there remains ample room for advancement in mimicking the intricacies of natural tissues and earning the confidence of the broader manufacturing community. In recent years, bio-implants have borne witness to a breathtaking array of advancements. From the development of biocompatible materials to the integration of 3D printing and nanotechnology, the field has experienced an upsurge in innovative approaches. Figure 1 depicts the global market trends of bio-implants, emerging challenges, potential demands, and constraints.

The primary goal of this Research Topic is to create a platform for in-depth discussions on cutting-edge concepts and innovative approaches aimed at enhancing the quality and commercial viability of implants. The collected articles revolve around advanced alloys and their manufacturing processes, the development of new resorbable bone wax formulations, innovative surface treatments, and coatings. Within this Research Topic, you will find five carefully selected papers contributed by 28 researchers, addressing a crucial aspect of sustainable implants, including novel biocompatible alloy materials, new coatings, and surface structure modification. These advancements hold the power to not only amplify the functionality of bio-implants but also to elevate the quality of patient outcomes.

## Resorbable bone wax development

The paper entitled “*Novel resorbable bone wax containing β-TCP and starch microspheres for accelerating bone hemostasis and promoting regeneration*” addresses and alleviates the numerous complications that may evolve due to the traditional non-resorbable bone wax used in clinical surgery. The researchers developed a novel resorbable bone wax using alkylene oxide copolymers, β-tricalcium phosphate, and starch microspheres to enhance bone regeneration and hemostasis. This novel resorbable bone wax shows high potential for clinical translation, offering a safer alternative to traditional bone wax.

## Coatings and surface modification

Surface engineering of bio-implants plays a significant role in sustainable implants. The paper entitled “*Nano selenium-doped TiO*_*2*_ *nanotube arrays on orthopedic implants for suppressing osteosarcoma growth*” revealed that the traditional metal implants used in osteosarcoma, a common malignant bone tumor, require reconstructive surgery, lack anti-tumor properties, which may potentially increase the risk of cancer recurrence. The researchers developed a selenium-doped TiO_2_ nanotube array film that modifies the implant surface and inhibits cancer cell growth while maintaining biocompatibility.

## Bulk nanostructured implant

The study entitled “*Nanostructured Ti-13Nb-13Zr alloy for implant application—material scientific, technological, and biological aspects*” investigated the production of advanced dental implants. The second generation alloy (Ti-13Nb-13Zr) underwent thermo-mechanical treatments, including equal channel angular swaging, recrystallization, and aging, resulting in a nanostructured microstructure with promising mechanical properties. These nanostructures demonstrated reduced bacterial biofilm formation and increased osteoblast cell proliferation compared to traditional titanium alloys.

3 D-Printing and Patient-Specific Implants: The paper entitled “*A 3D-printed patient-specific modular implants for pelvic reconstruction of bone tumors involving the sacroiliac joint*” investigates the impact of 3D-printed patient-specific implant reconstruction, and demonstrates its remarkable stability and minimal risk of failure. The study reported satisfactory surgical outcomes for all Six patients, achieving good functional results and osseointegration of the implant to the ilium and sacrum. In the study entitled “*Laser powder bed fusion (LPBF) of commercially pure titanium and alloy development for the LPBF process*”, the researchers tuned the LPBF process parameter by using a statistical technique to establish a set of optimized process parameters and a process window for LPBF printing of small commercially pure (CP) titanium parts with minimized volume porosity. In this research work, the phase, texture, and microstructure analyses have suggested that the composition Ti-0.44O-0.5Fe-0.08C-0.4Si-0.1Au-0.1B-0.1Y holds great promise for reducing anisotropy in Laser Powder Bed Fusion (LPBF) processing. However, to fully harness its potential, further investigations into LPBF printing techniques and the formation of Y2O3 are critical.

## Conclusion

The field of bio-implants is poised for exponential growth and transformation. Despite the significant progress made in bio-implants, several challenges persist, including cost-effectiveness, accessibility, and long-term performance. Further research and clinical validation are essential to realize their full potential in enhancing healthcare. The future of bio-implants lies in interdisciplinary collaboration, continuous research, and a focus on personalized medicine.

